# Patients with Achilles Tendon Rupture Have a Degenerated Contralateral Achilles Tendon: An Elastography Study

**DOI:** 10.1155/2018/2367615

**Published:** 2018-12-06

**Authors:** Qianru Li, Qi Zhang, Yehua Cai, Yinghui Hua

**Affiliations:** ^1^Department of Sports Medicine, Huashan Hospital, Fudan University, Shanghai, China; ^2^Institute of Biomedical Engineering, Shanghai University, Shanghai, China; ^3^Institute for Advanced Communication and Data Science, Shanghai University, Shanghai, China; ^4^Department of Ultrasound, Huashan Hospital, Fudan University, Shanghai, China

## Abstract

**Purpose:**

To evaluate differences of Achilles tendon (AT) hardness and morphology between asymptomatic tendons in patients with acute AT ruptures on the contralateral side and asymptomatic tendons in healthy people by using computer-assisted quantification on axial-strain sonoelastography (ASE).

**Methods:**

The study consisted of 33 asymptomatic tendons in 33 patients (study group) and 34 tendons in 19 healthy volunteers (control group). All the tendons were examined by both ASE and conventional ultrasound. Computer-assisted quantification on ASE was applied to extract hardness variables, including the mean (Hmean), 20^th^ percentile (H20), median (H50) and skewness (Hsk) of the hardness within tendon, and the ratio of the mean hardness within tendon to that outside tendon (Hratio) and three morphological variables: the thickness (THK), cross-sectional area, and eccentricity (ECC) of tendons.

**Results:**

The Hmean, Hsk, H20, H50, and Hratio in the proximal third of the tendon body in study group were significantly smaller than those in control group (Hmean: 0.43±0.09 vs 0.50±0.07, p=0.001; Hsk: -0.53±0.51 vs -1.09±0.51, p<0.001; H20: 0.31±0.10 vs 0.40±0.10, p=0.001; H50: 0.45±0.10 vs 0.53±0.08, p<0.001; Hratio: 1.01±0.25 vs 1.20±0.23, p=0.003). The THK and cross-sectional area of tendons in the study group were larger than those in the control group (p<0.05).

**Conclusions:**

As a quantitative objective method, the computer-assisted ASE reveals that the asymptomatic ATs contralateral to acute rupture are softer than those of healthy control group at the proximal third and the asymptomatic tendons in people with rupture history are thicker, larger, and rounder than those of normal volunteers especially at the middle and distal thirds of AT body.

## 1. Introduction

The Achilles tendon (AT) is the thickest and strongest tendon in the human body. Originating from the aponeuroses of the gastrocnemius and soleus muscles and being inserted at midsection of the posterior calcaneal tuberosity, the Achilles tendon is a part of the musculotendinous unit that spans three joints, producing knee flexion, tibiotalar flexion, and subtalar inversion [1]. A rupture of the Achilles tendon is a disruption of the Achilles tendon and usually occurs during high-impact sports. Given the critical function of AT, the rupture of AT can be a devastating event for general population and a career threatening injury for athletes as one-thirds of the national sports players who suffered an Achilles tendon rupture could never play at the same sports level again [2]. With the increase of leisure time and activity in recreational sports, the incidence of overuse injuries and acute sports injuries are growing as well [3]. Despite its strength, the Achilles tendon is one of the most frequently injured tendons in the human body [4], and the incidence of AT ruptures is increasing over the past decades [5, 6]. An interesting finding is that the risk of contralateral tendon rupture is increased in individuals with a previous Achilles tendon rupture [7]. Thus, we suppose that the quality of an asymptomatic Achilles tendon in people suffering contralateral rupture previously is different from that of healthy control group.

Degenerative alterations of biomechanical properties such as hypoxic degenerative tendinopathy, mucoid degeneration, tendolipomatosis, and calcifying tendinopathy were found in a study that included 397 Achilles tendon biopsy specimens from patients with spontaneous AT ruptures [4]. These histopathological changes may alter the physical properties of Achilles tendons. As one of the physical properties of materials, hardness is a general term describing the ability of a material to resist plastic deformation. And the hardness can be represented as strains quantitatively [8].

Axial-strain sonoelastography (ASE) is a newly developed technique of ultrasonography for measurement of tissue hardness, which involves manual axial compression of tissue using the hand-held ultrasound (US) transducer to generate tissue strains (deformations) [9, 10]. The applications of ASE on imaging of Achilles tendon disease have increased in recent years [11–13], and the accuracy of its measurement has been extensively validated with phantoms and excised tissues [8, 14].

Previous research employed various elastography grading systems for qualitative identification of tendon disorders and the elasticity grades were generally scored by experienced radiologists via visual assessment, which means that the assessment was intuitionistic and no objective values were extracted [13, 15, 16]. Thus, the judgement and diagnoses based on such methods were too subjective to attain ideal repeatability even in terms of intraobserver, let alone in the point of interobserver. For these reasons, a more objective method is needed urgently. To handle this, we design a computer-assisted quantification program to obtain objective tendon hardness values in order to improve the accuracy and repeatability.

To our knowledge, there were hardly any studies reporting different hardness patterns between asymptomatic tendons in patients with contralateral AT rupture (study group) and asymptomatic tendons in normal volunteers (control group). Therefore, the purpose of this study was to propose the computer-assisted method for quantifying tendon hardness with ASE and apply it to comparing asymptomatic contralateral Achilles tendons in patients suffering acute ruptures previously with those of normal volunteers. Our hypothesis is that the quality of asymptomatic contralateral Achilles tendons was softer than asymptomatic tendons of healthy control group.

## 2. Materials and Methods

### 2.1. Study Population

The study was approved by the institutional review board of Huashan Hospital, and all participants' informed consent was obtained. All patients were prospectively included between June 2014 and November 2017.

The inclusion criteria for normal volunteers (control group) were (1) aged over 18 years and (2) willing to join in this research. The exclusion criteria for volunteers were pregnancy, history of AT rupture or AT surgery, pain in the AT, and morphologic abnormalities at US (B-mode and/or power Doppler). Volunteers with a history of systemic, metabolic, or endocrine diseases; and those treated with corticosteroids, estrogens, quinolones, and cholesterol drugs were excluded because of known associations between those factors and tendon diseases [17].

The inclusion criteria for the study group were (1) the intact ATs of patients with contralateral AT ruptures and the ruptures were confirmed by surgery; (2) patients aged over 18 years; (3) patients willing to join in this research. The exclusion criteria were patients with histories of tendon disorders such as pain, swelling, and dysfunction in the asymptomatic AT.

All people of these two groups were classified by a sports medicine surgeon with 19 years of clinical experience. And in total, 33 asymptomatic Achilles tendons in 33 patients (27 male and 6 female) were included in study group and 34 ATs in 19 normal volunteers (14 male and 5 female) were included in control group. Among the 19 people in control group, 4 tendons were excluded because of acute lateral ankle ligament injuries. While for these 4 patients, the included ATs were confirmed intact with the help of careful physical examination and traditional ultrasound examination.

### 2.2. Ultrasound Imaging Acquisition

All people were examined in a prone position with the foot hanging over the edge of the examination bed by the HI VISION Ascendus system (Hitachi Medical, Tokyo, Japan) equipped with a linear array transducer (EUP-L75. 5-18MHz). Both the conventional US and the ASE imaging were performed on ATs by a senior radiologist with 14 years' experience in musculoskeletal US.

The bodies of AT were divided length-equally into three thirds for the convenience of further analysis: (1) proximal (near to musculotendinous junction), (2) middle, and (3) distal (near to insertion site). All included tendons underwent ASE and conventional US for each third.

**Figure 1:**
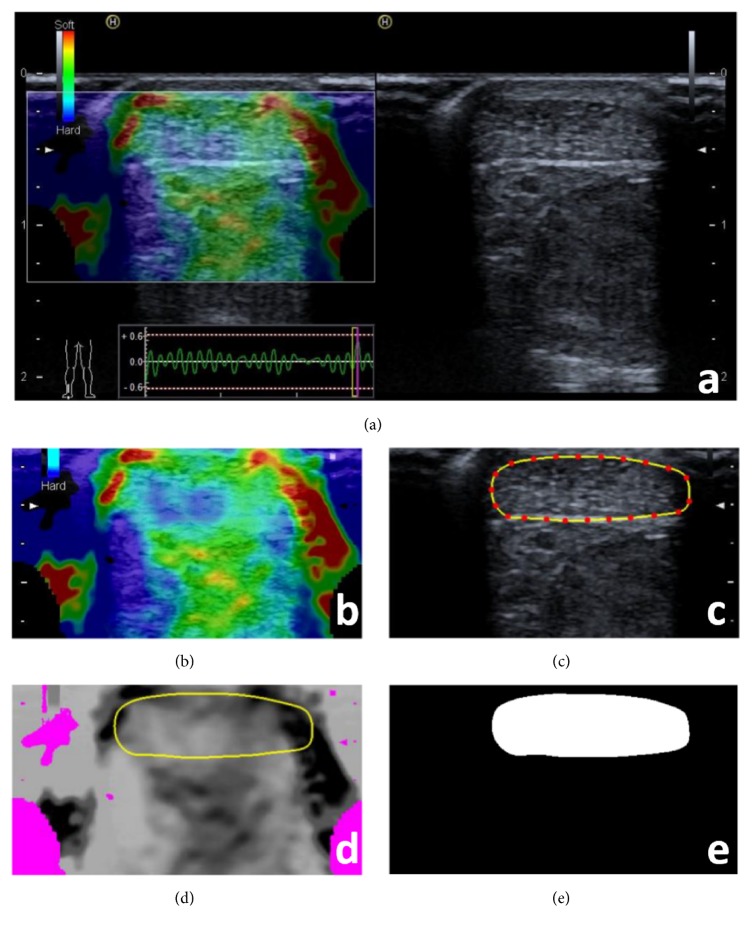
An example for illustrating the computer-assisted quantification of tendon hardness on axial-strain sonoelastography (ASE). The imaging system provides dual-modality visualization in a full screen consisting of two parts: the right part depicts a greyscale B-mode image and the left is a composite colour image that is a pure colour elastogram overlaying the same B-mode image (a). The pure colour elastogram (b) is retrieved by subtracting the B-mode image from the composite image. The tendon borders are delineated by using interactive software (c). The pure colour elastogram is transformed to a greyscale image (d). The location of the tendon is depicted in a binary template (e).

In the process of examination, light repetitive compression was exerted over an AT with the hand-held transducer. Perpendicular to the AT, the pressure was applied vertically and was adjusted according to the real-time feedback visual indicator of pressure on the screen to depict a sinus curve [18], as shown in lower left of Figure 1(a). The elastograms were presented in colour overlaying the B-mode greyscale image, using a colour map from red to blue indicating low to high hardness (Figure 1(a)).

The sinus curve should be stable at an almost constant amplitude and frequency. For each scan plane, a representative image was selected from cine loops and stored in DICOM format for further image processing including computer-assisted quantification. A representative image should display tendon boundaries and regular structured peritendinous layers including paratenon, muscles, and bursae, so as to ensure tissue movement not interfering with intratendinous colouring [19].

### 2.3. Computer-Assisted Quantification of Tendon Hardness

A computer-assisted quantification program was designed to obtain tendon hardness via MATLAB R2007a (MathWorks, Natick, MA, USA). The program was conducted by the same radiologist who had manipulated the scans.

The Hitachi ASE system provides dual-modality visualization in a full screen (Figure 1(a)). The right part is a grey-scale B-mode image, while the left part is a composite colour image represented as the red-green-blue (RGB) format superimposed on the greyscale B-mode image. The pure colour elastogram in Figure 1(b) was acquired by subtracting the B-mode image from the composite image, but still in RGB format. The hardness of examined tissue was then reconstructed by calculating the hue value from the pure elastogram [20], ranging from 0 (red, softest) to 5/6 (blue/hardest). The areas appearing as black holes and black shades on ASE colour images (Figures 1(a) and 1(b)) were excluded from further calculation and analysis in the greyscale image transformed from the RGB image.

Software was designed for interactive delineation of a tendon border on either the B-mode greyscale image or the ASE colour image, and it would automatically map the outlined contour back to the other image simultaneously. The interactions between a user and the system were as simple as clicks on either image while selecting several points on the tendon border (red dot in Figure 1(c)), and the B-spline interpolation of the discrete points was employed to get a smooth closed curve (yellow curves in Figures 1(c) and 1(d)) [21]. The location of the tendon was depicted in a binary template (while ellipse in Figure 1(e)).

The hardness of tendons was quantified by calculating the statistics of tendon hue values on ASE image, including the mean (Hmean), skewness (Hsk), 20^th^ percentile (H20) and 50^th^ percentile (median; H50) of the hue values within tendon, and the ratio of the mean hardness within tendon to that outside tendon (Hratio). Here, Hsk value indicates the probability of asymmetry distribution of tendon hardness. The more negative the Hsk is, the harder the tendon is.

Morphological parameters of the tendons including the thickness (THK), cross-sectional area, and eccentricity (ECC) were also computed automatically. ECC ranges from 0 to 1, and a larger ECC represents a more oblate shape of tendon. The more the value approaches 0, the rounder the cross-sectional is [19].

### 2.4. Statistical Analysis

The sample sizes were calculated based on the criteria of *α*=0.05 and *β*=0.80 and the minimal sample sizes for all the variables were 24 in both groups [22]. Thus in this study, the sample sizes of 33 in the study group and 34 in the control group were valid enough.

Data analysis was performed using SPSS 24.0 software (IBM Corp, Armonk, New York, USA). Patient age was expressed as median and range because of its nonnormal distribution. The body mass index (BMI), tendon hardness, and morphological parameters were expressed as mean ±SD because they were normally distributed via the Lilliefors test.

The differences of gender and included side between two groups were assessed with the Chi-square test and the difference of age was assessed with the Kruskal–Wallis test. BMI and variables of tendon thirds were compared by using unpaired two-sample t tests between two groups, i.e., study group and control group. Quantitative parameters calculated on transversal planes were compared between groups.

An overall p value of less than 0.05 was considered to indicate a significant difference.

## 3. Results

All the original data could be found in the supplementary materials and the analysis results are as follows.

### 3.1. Patient Demographics

There were 33 patients (27 male and 6 female) with a median age of 35 (range 25-65) in the study group and 19 normal volunteers (14 male and 5 female) with a median age of 35 (range: 27-58) in the control group. The average body mass index (BMI) in the study group was 23.64±2.84 kg/m^2^ and that in the control group was 23.81±2.15 kg/m^2^. A total of 33 ATs (13 left and 20 right) in 33 patients and 34 ATs (16 left and 18 right) in 19 normal volunteers were included in the study group and control group, respectively. There was no significant difference between the two groups in terms of gender, age, BMI and included tendons (Table 1).

### 3.2. Comparisons of Hardness and Morphology between Groups

At transversal scans, Hmean, H20, H50, Hsk, and Hratio in the proximal third of the tendon body in study group were significantly smaller than those in control group (Hmean: 0.43±0.09 vs 0.50±0.07, p=0.001; Hsk: -0.53±0.51 vs -1.09±0.51, p<0.001; H20: 0.31±0.10vs 0.40±0.10, p=0.001; H50: 0.45±0.10 vs 0.53±0.08, p<0.001; Hratio: 1.01±0.25 vs 1.20±0.23, p=0.003) (Table 2), indicating that the proximal third tendons in the study group were softer than those in the control group. No significant differences were found between two groups when the middle and distal thirds were taken into consideration (Table 2).

Compared with the asymptomatic tendons in normal volunteers, two morphological variables (thickness and cross-sectional area) of asymptomatic tendons in rupture population were larger (THK at three thirds: 0.45±0.09 vs 0.41±0.06 0.53±0.13 vs 0.45±0.04; 0.47±0.09 vs 0.39±0.05; Area at three thirds: 0.57±0.16 vs 0.50±0.12; 0.68±0.25 vs 0.56±0.10; 0.83±0.27 vs 0.63±0.11), and the eccentricity in study group was closer to 0 (0.95±0.02 vs 0.96±0.01; 0.93±0.03 vs 0.95±0.01; 0.97±0.01 vs 0.98±0.01), revealing that the tendons from study group were thicker, larger, and rounder than those from healthy control group especially in the middle (p=0.002, 0.018, and 0.001, respectively) and distal thirds (p<0.001, <0.001, and =0.035, respectively) (Table 3).

## 4. Discussion

In the current study, the most important finding is the hardness of asymptomatic contralateral Achilles tendons in patients with acute AT ruptures is significantly lower than that of normal volunteers at the proximal thirds by applying a set of quantitative variables derived from ASE with computer-assisted methods.

Kannus et al. found that, in most of the spontaneously ruptured tendons, the pathological changes via biopsy were degenerative such as hypoxic degenerative tendinopathy, mucoid degeneration, and tendolipomatosis [4]. Compared with the normal tendons, the fat metabolism and inflammatory cells of the tendons suffering from acute or chronic ruptures were detectable [23]. With the elastography technique, De Zordo et al. found that a normal tendon was hard, and 57% of tendons with symptoms of tendinopathy showed marked softening [11]. Chen et al. demonstrated significant softening of the completely ruptured ATs compared with the normal tendons [24]. With the process of healing after Achilles tendon ruptures, the elastography showed that the ATs became harder and harder to approach the hardness of normal tendons. Furthermore, the increasing hardness obtained by US elastography revealed correlation with decreasing Bonar scale under microscope [23], indicating less pathological changes [25]. Thus, we suspected that the decreasing of hardness uncovered by ASE in asymptomatic tendons may be a detectable sign of the early lesion. Although the tendons contralateral to ruptured ATs were asymptomatic and intact clinically, the decreasing hardness revealed by elastographies indicated that bilateral pathological changes existed in patients with unilateral AT ruptures histories. This finding provided us with more clues about AT pathology and the possibility to supervise the qualities of ATs in the early stage so that the preventive measure could be taken to avoid rupturing.

Lots of studies have reported that, at middle of the free tendon, the “critical zone,” the AT was most vulnerable to injury [26]. We know that the AT, with the length of 15 cm, is the largest tendon in the human body. Thus, the description of “middle part” of AT in prior articles is a very broad range. In this study, the obvious hardness differences firstly occurred at the proximal third of the AT body. These results might be an early and more detailed indicator for revealing the specific location that pathological changes of Achilles tendon appeared.

Another important finding in this study is that the ATs on the asymptomatic side in patients with acute AT ruptures previously were thicker and larger than those in healthy control group at the middle and distal thirds of AT bodies. S. Rosager et al. stated that the chronic exposure to repetitive loading would result in the enlargement of cross-sectional areas of Achilles tendons [27]. In this study, the alterations of areas at the middle and distal thirds may be the outcomes of these compensatory adaptations. While point-in-time when the changes occurred was unknown, so it could not be clarified whether the morphological alteration was the contemporaneous changes accompanying the development of the AT ruptures or the adaptation outcomes of overload and overuse due to the contralateral strength defect caused by AT ruptures. In addition, Birk D.E et al. found during the process of tendon development that an inverse relationship between the amount of type III collagen and fibril diameter may exist [28]. The changes in diameters found in our study may also reveal the existence of microtrauma and healing procedure in asymptomatic tendons contralateral to acute rupture, though they were intact clinically.

The male-to-female ratio has been reported by most authors in the range of 4.4:1 to 9.5:1 in the previous studies [5, 29–31]. And the ratio in this study (4.5:1) was in accordance with the previous ones. The possible reasons for the high incidence in male were greater prevalence of males than females who are involved in sports and the differences of properties in ATs among male and female, although there may be other as yet unrecognized factors. Given that the sample size was relatively small, the stratification analysis was improper to apply to clarify these speculations. The BMI and age could also impose effect on AT ruptures [27], and in our studies those two variables showed no difference between the study and control groups, which means that the bias led by age and BMI was balanced.

There are several limitations in the present study. Firstly, the ASE is highly operator-dependent. The hardness obtained from ASE was a relative value instead of an absolute one. In other words, the elasticity was determined by the selection of range of in interest. Although in this study, many details of operation were strictly controlled, a more objective method, such as shear-wave elastography [32], should be applied to verify the findings. Secondly, this study utilized the transversal images alone. The longitudinal images are supposed to be included for analysis in the further research to complement the findings. Thirdly, even in the normal people, there were still a large proportion (62%) tendons showed soft areas [33]. To be specific, even if we regarded the asymptomatic tendons in normal volunteers as control group, the conclusion could only be restricted to the comparison between the asymptomatic tendons of patients with previous ruptures and asymptomatic tendons in normal population rather than the normal tendons. At last, although the relationship between hardness reflected by elastographies and pathologic changes has been verified in previous studies, the exact factors changing the hardness needed clarifying in the future.

Now, the computer-assisted quantification method was still in the optimization and intelligentization process and has not been commercially available, while ASE combined with computer-assisted quantification is recognized as a feasible technique in the assessment of AT ruptures, providing quantitative information about elastic properties of tendons. This quantitative hardness information would be potentially valuable for differentiating the qualities of tendons and predicting the rupture risk of asymptomatic tendons in patients with histories of acute AT ruptures. Thus, this inexpensive and real-time technique might be useful for monitoring the pathological progression of AT ruptures and for evaluating the healing process after a conservative or surgical therapy in the near future.

## 5. Conclusions

It is shown that, in patients with AT ruptures, the asymptomatic ATs contralateral to ruptured tendon are softer than those of healthy control group at the proximal third, and the asymptomatic tendons are thicker, larger, and rounder than those of healthy control group, especially at the middle and distal thirds of AT body. The ASE could act as a potentially valuable tool in daily clinical practice to provide important information of the early stage lesions, which is crucial for predicting the ruptures of ATs and rendering protective suggestions in advance.

## Figures and Tables

**Table 1:** Demographic data.

Variable	Study group	Control group	p value
Gender (male/female)	27 / 6	14 / 5	0.726
Age (years)	35 (25-65)	35 (27-58)	0.493
BMI (kg/m^2^)	23.64±2.84	23.81±2.15	0.900
Included tendon (left/right)	13 / 20	16 / 18	0.624

**Table 2:** Comparisons of hardness between the study group and control group.

Third		Hmean	Hsk	H20	H50	Hratio
Proximal	Study	0.43±0.09	-0.53±0.51	0.31±0.10	0.45±0.10	1.01±0.25
	Control	0.50±0.07	-1.09±0.51	0.40±0.10	0.53±0.08	1.20±0.23
	p value	0.001	<0.001	0.001	<0.001	0.003
Middle	Study	0.42±0.06	-0.46±0.42	0.31±0.08	0.44±0.07	0.97±0.19
	Control	0.45±0.07	-0.59±0.33	0.33±0.07	0.47±0.08	1.05±0.23
	p value	0.181	0.146	0.243	0.103	0.148
Distal	Study	0.43±0.07	-0.36±0.46	0.28±0.09	0.44±0.09	0.91±0.14
	Control	0.43±0.05	-0.22±0.35	0.27±0.06	0.44±0.08	0.91±0.13
	p value	0.793	0.174	0.767	0.979	0.959

**Table 3:** Comparisons of morphological variables between the study group and control group.

Third		THK (cm)	Area (cm^2^)	ECC
Proximal	Study	0.45±0.09	0.57±0.16	0.95±0.02
	Control	0.41±0.06	0.50±0.12	0.96±0.01
	p value	0.047	0.058	0.132
Middle	Study	0.53±0.13	0.68±0.25	0.93±0.03
	Control	0.45±0.04	0.56±0.10	0.95±0.01
	p value	0.002	0.018	0.001
Distal	Study	0.47±0.09	0.83±0.27	0.97±0.01
	Control	0.39±0.05	0.63±0.11	0.98±0.01
	p value	<0.001	<0.001	0.035

## Data Availability

All the original ultrasonograms were saved in the radiology information system of our hospital and could be retrieved by the name of each person. The primary data extracted from the original ultrasonograms were submitted as supplementary materials and were presented as an EXCEL version.

## Supplementary Materials

Supplementary Materials The Achilles tendon (AT) was divided into three parts to analyze. The first SHEET showed the proximal part of the AT and the SHEET 2 and SHEET 3 represented the middle and distal part, respectively. In each SHEET, the first COLUMN showed the name of each person and the second one to the sixth one represented the gender (男: male; 女: female), age, included at the side of AT (L: left; R: right), part of the AT (P: proximal; M: middle; D: distal), and group (A: asymptomatic; N: normal), respectively. The following COLUMN was the various kinds of parameters about hardness and morphology of AT that we are concerned about.
